# Machine learning based identification of relevant parameters for functional voice disorders derived from endoscopic high-speed recordings

**DOI:** 10.1038/s41598-020-66405-y

**Published:** 2020-06-29

**Authors:** Patrick Schlegel, Stefan Kniesburges, Stephan Dürr, Anne Schützenberger, Michael Döllinger

**Affiliations:** Department of Otorhinolaryngology, Division of Phoniatrics and Pediatric Audiology, University Hospital Erlangen, Friedrich-Alexander-University Erlangen-Nürnberg, Erlangen, Germany

**Keywords:** Scientific data, Diseases

## Abstract

In voice research and clinical assessment, many objective parameters are in use. However, there is no commonly used set of parameters that reflect certain voice disorders, such as functional dysphonia (FD); i.e. disorders with no visible anatomical changes. Hence, 358 high-speed videoendoscopy (HSV) recordings (159 normal females (N_F_), 101 FD females (FD_F_), 66 normal males (N_M_), 32 FD males (FD_M_)) were analyzed. We investigated 91 quantitative HSV parameters towards their significance. First, 25 highly correlated parameters were discarded. Second, further 54 parameters were discarded by using a LogitBoost decision stumps approach. This yielded a subset of 12 parameters sufficient to reflect functional dysphonia. These parameters separated groups N_F_ vs. FD_F_ and N_M_ vs. FD_M_ with fair accuracy of 0.745 or 0.768, respectively. Parameters solely computed from the changing glottal area waveform (1D-function called GAW) between the vocal folds were less important than parameters describing the oscillation characteristics along the vocal folds (2D-function called Phonovibrogram). Regularity of GAW phases and peak shape, harmonic structure and Phonovibrogram-based vocal fold open and closing angles were mainly important. This study showed the high degree of redundancy of HSV-voice-parameters but also affirms the need of multidimensional based assessment of clinical data.

## Introduction

In the field of laryngology, high-speed videoendoscopy (HSV) is an assessment technique about to be established in clinics^1,2^. This technique is already commonly used in research settings and large clinics to investigate the oscillations of the vocal folds in the larynx, forming the basis signal for our voice^3–5^.

During voice production an airstream rises from the lungs through the trachea and sets the vocal folds in motion. Vibrating at oscillation frequencies between 150 and 400 Hz^6^, the vocal folds divide the continuous airstream in a series of flow pulses producing the fundamental frequency and basis signal of the voice. The flow pulses are then further modulated by the vocal tract, tongue and lips producing audible voice and speech^7,8^. However, during singing the vocal folds can vibrate much faster. Oscillation frequencies of up to 1568 Hz with complete glottal closure are reported^9^.

In general, periodic and symmetric vocal fold oscillations with complete glottal closure indicate a healthy voice^10–12^. Respectively asymmetric, aperiodic oscillations or a large continuously open part of the glottis indicate a voice disorder^13–15^. Different systems exist to classify voice disorders, such as subdivisions in central and peripheral dysphonias; neurogenic, psychogenic and myogenic dysphonias or mucosal and neuromuscular disorders^16^. In this work, the European classification in organic and functional voice disorders will be used since only healthy subjects and subjects suffering from functional dysphonia (FD) were investigated.

FD is a diagnosis of exclusion meaning that the subject has no organic voice disorders i.e. visible changes in the vocal tract or injuries of the vocal folds^17^. Symptoms of FD include hoarseness, changes in pitch or other changes in voice quality^16^. Also, purely psychologic causes are in the range of possibilities^18^.

The current “gold standard” in clinics to investigate vocal fold oscillations and respectively voice disorders is stroboscopy^19–21^. However, stroboscopy only produces an artificial slow motion perspective of the vocal fold vibration and therefore data that cannot be interpreted in the case of irregular vibrations; HSV does not have this disadvantage^22^.

**Figure 1:**
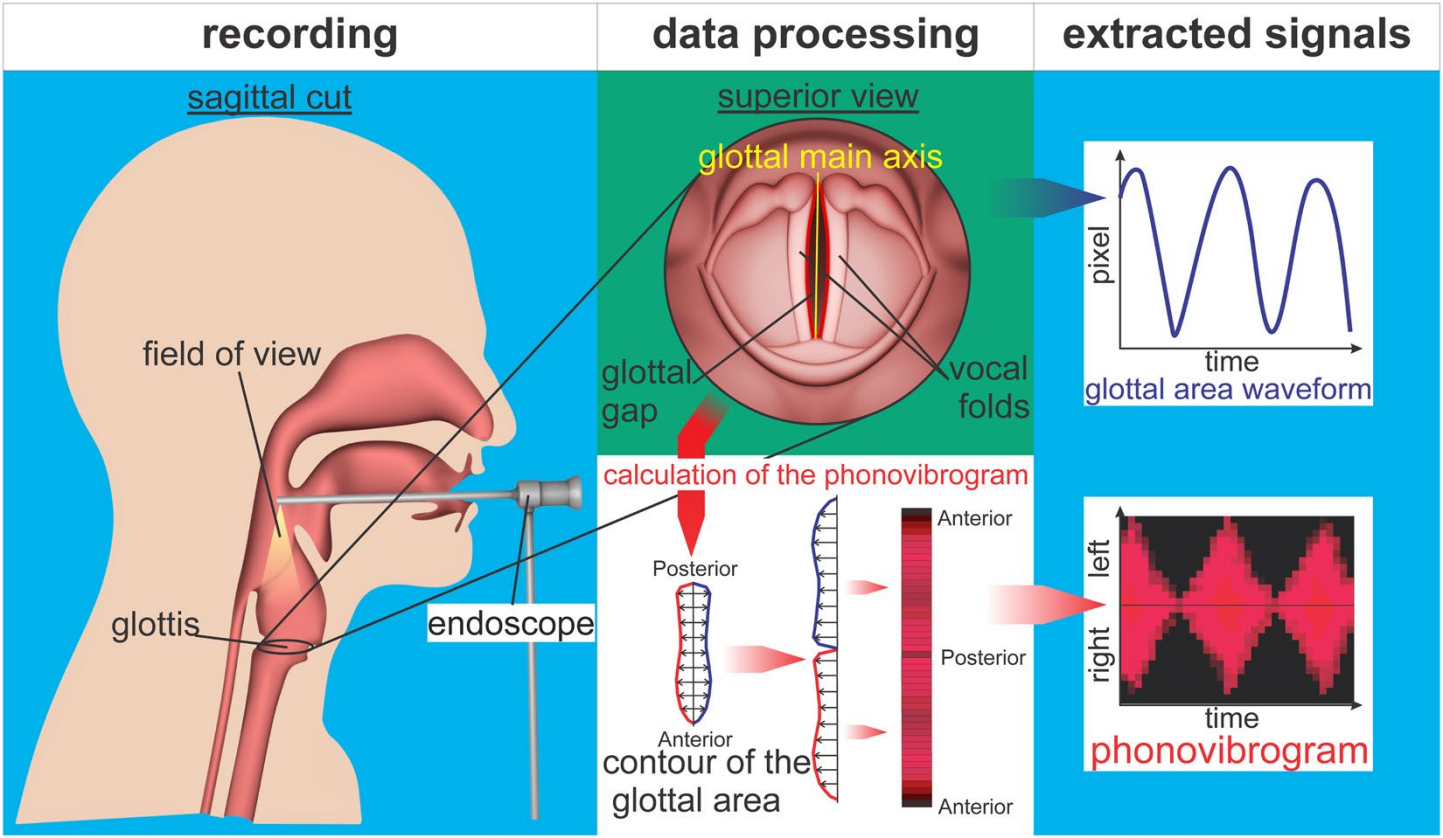
HSV recording using a rigid endoscope yielding 1D-GAW and 2D-PVG signals.

As depicted in Fig. 1, during HSV recording, a rigid endoscope is inserted in the throat of the subject recording the vocal folds from above. The fast oscillations of the vocal folds are recorded with sampling rates of about 4000 Hz severely exceeding the oscillation frequencies of the vocal folds^22^. Based on the recorded video data the 1D Glottal Area Waveform (GAW) that represents the area between the vocal folds (the “glottal area”) over time can be computed; i.e. the GAW is the function of glottis-pixels over time (see Fig. 1, top right). Another (2D) signal determinable from HSV-recordings is the Phonovibrogram (PVG) introduced by Lohscheller *et al*.^23^. The PVG depicts the whole oscillation pattern of the contour of the glottal area over time in one image, as shown in Fig. 1, bottom right.

From these signals different kinds of GAW- and PVG-based parameters are calculable^24^.

In recent years machine learning based approaches have grown in popularity in voice research^25–27^. Machine learning was also used in combination with parameters to separate healthy from disordered voices^28–30^. Callan *et al*. trained a self-organizing map using acoustic parameters to differentiate normal from disordered voices and achieved an overall accuracy of 0.76^28^. Awan and Roy achieved 0.75 accuracy for separation of normal, breathy, rough and hoarse voices also using acoustic parameters^29^. PVG based parameters were used by Voigt *et al*. to differentiate normal and FD voices with 0.81 accuracy^30^. Also a few more recent studies were published reporting accuracies of up to 1.00 using only acoustic measures^31,32^. However, because of the perfect accuracies stated, the reliability of these findings may be questionable.

It is known that many features associated with FD (for instance incomplete glottis closure) also frequently occur in healthy subjects^33^. This indicates that multidimensional approaches applying different parameters are needed to separate healthy and disordered subjects. Furthermore, many parameters describing laryngeal features are redundant^5,34^. However, the redundancies of parameters are not yet fully explored, and it is not known which parameters best characterize FD or other voice disorders. For this reason, this study uses a multidimensional approach investigating GAW- and PVG-based parameters in regard of their linear dependencies and expressiveness in differentiating healthy and disordered voices. The aims of this work are:

1. Determine linear relations between a large set of parameters using clinical data and discard redundant parameters.
2. Investigate GAW- and PVG-based parameters and which combination of them is best suited for separating healthy from FD subjects.
3. Discuss why the final parameter set is able to differentiate groups and which features of the vocal fold oscillation process are described by these parameters.

## Methods

358 HSV recordings of 260 female and 98 male subjects were investigated. The recordings were taken using a 70° rigid endoscope attached to a clinically used Photron Fastcam MC2 camera (frame rate: 4000 fps, resolution: 512×256 pixels). All subjects phonated the vowel /i/ at a comfortable (i.e. habitual) pitch and loudness level (sustained phonation) and all recordings had a length of at least 250 ms. The study was approved by the ethic committee of the Medical School at Friedrich-Alexander-University Erlangen-Nürnberg (no. 290_13B) and all methods were carried out in accordance with relevant guidelines and regulations. Written consent was obtained by the subjects. Recordings of females and males were each subdivided into one healthy group and one disordered group:

- Recordings of healthy subjects with normal sounding voices (Females: N_F_, Males: N_M_).
- Recordings of disordered subjects before treatment (Females: FD_F_, Males: FD_M_).

Disordered patients were diagnosed by our clinicians. All disordered patients have only FD with no concurrent organic disorders. Table 1 contains the numbers of recordings from female and male subjects in healthy (N_F_, N_M_) and disordered (FD_F_, FD_M_) groups. For each subject one HSV-recording was performed.

**Table 1:** Number of HSV-videos in healthy and disordered groups.

	Healthy	Disordered
Females	159 (N_F_)	101 (FD_F_)
Males	66 (N_M_)	32 (FD_M_)

### Segmentation of the glottal area

**Figure 2:**
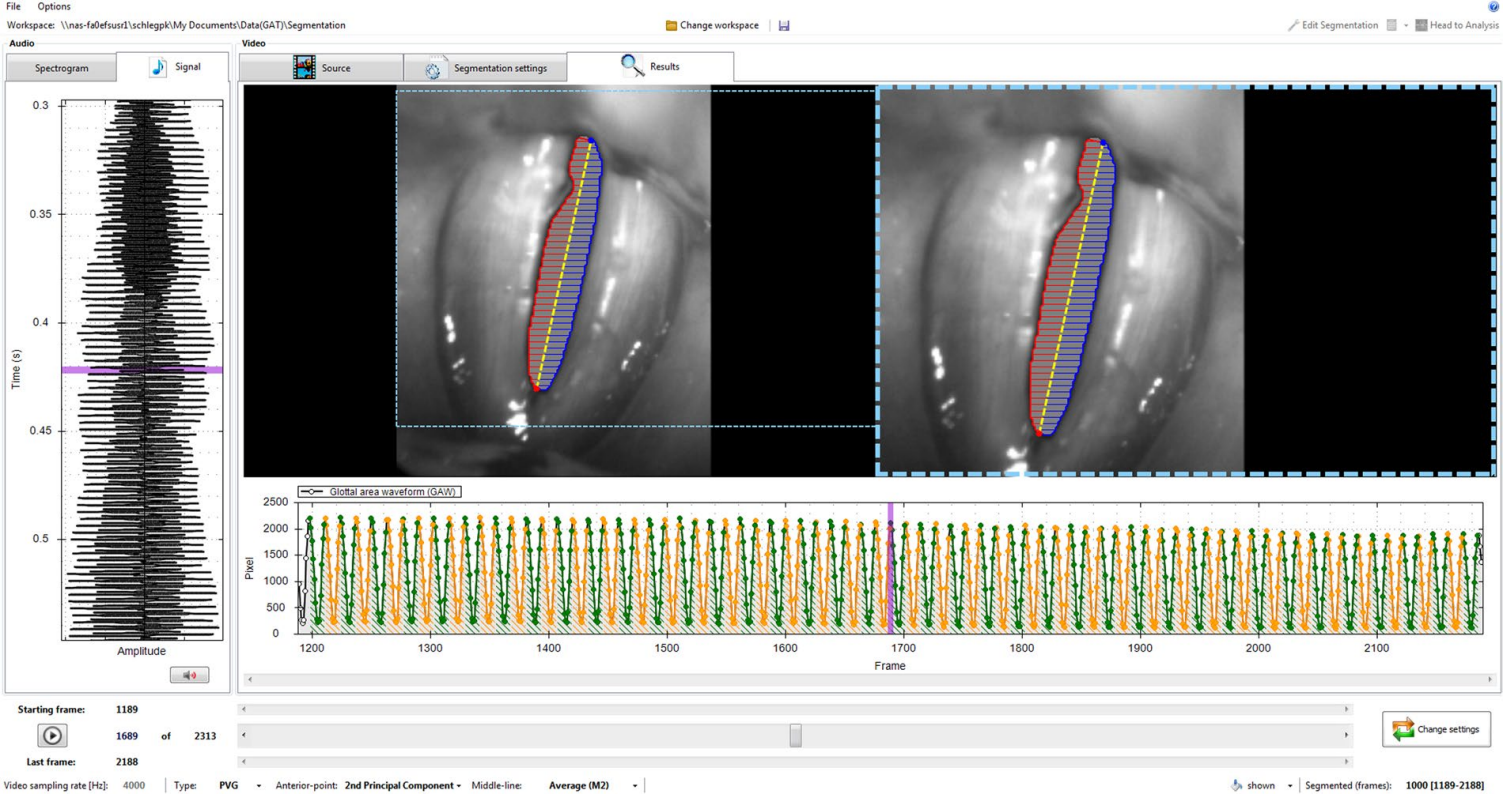
Glottis segmentation using Glottis Analysis Tools.

The glottal area of the collected videos was segmented using an in house developed version of Glottis Analysis Tools (GAT-2018). At the moment, GAT is used by 27 voice groups in 7 countries. A screen shot of GAT featuring glottis segmentation is depicted in Fig. 2.

**Figure 3:**
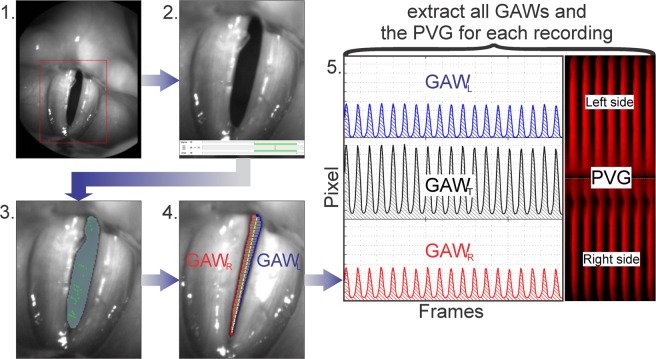
Segmentation Process. 1. Selection of the glottis region; 2. Selection of a 1000 frames section; 3. Segmentation of the glottal area; 4. Calculation of the partial GAWs (GAW_L_ and GAW_R_) and 5. Extraction of all GAWs (GAW_T_, GAW_L_ and GAW_R_) and the PVG.

The segmentation process is illustrated in Fig. 3. A summary of the process is given here:

1. A section of the video containing the entire glottis region was selected.
2. A segment of 1000 frames (250 ms) of the video was selected during which the subject holds sustained phonation.
3. Seed points were chosen and the brightness thresholds were adjusted to segment the dark glottal area between the vocal folds.
4. The contour of the glottal area was calculated as described in^35^ and a midline was selected dividing the total glottal area in two sides. Left (GAW_L_) and right (GAW_R_) partial GAWs were computed for each side by numerical integration over the distances between the midline and the left and right contour lines.
5. The total GAW of the entire area (GAW_T_), GAW_L_ and GAW_R_ as well as the Phonovibrogram (PVG) for all 1000 segmented frames were extracted (for a detailed explanation of the PVG see^23^).

### Parameter computation

For each of the 358 recordings one GAW_T_, GAW_L_, GAW_R_ and PVG signal were calculated. Extremum based cycles were determined for the GAW_T_ signals and conferred to the PVG, GAW_L_ and GAW_R_. Then for each GAW_T,_ 41 parameters were computed. 18 symmetry parameters were calculated using GAW_L_ and GAW_R_ and further 32 parameters based on PVG. In the supplementary information in Table S1, names, abbreviations, sources and descriptions of all 91 parameters (starting parameter set: **HSV**_**0**_) are given. Parameters were calculated for maximum based cycles (i.e. each cycle starts at a sufficiently distinct local maximum and ends before the next distinct local maximum) with exception of PhA[Mean] PhA[StD], PhAI[Mean] and PhAI[Std] which required minimum based cycles (analogously to maximum based cycles but using local minima instead) by their definition. The following investigations were performed using MATLAB (version 9.3.0.713579, R2017b).

### Linear dependencies

In a first step the parameters were investigated for linear dependencies by calculating Pearson Correlation Coefficients (PCC) between all parameters over all healthy and disordered groups. Parameters being correlated “very high” (corr. ≥ 0.9 following the suggestions of Mukaka^36^) were removed. Furthermore, based on previous studies^5^, four additional parameters were removed that were only correlated “high” (0.9> corr. ≥ 0.7).

By calculating PCCs over all healthy and disordered groups, regardless of health status or gender, only correlations were found that were consistent for all cases i.e. correlated parameters behave strongly similar for all data. This implies that the parameters are redundant. For this reason based on the found PCCs, the parameter set **HSV**_**0**_ was reduced yielding parameter set **HSV**_**1**_.

### Influence of subject age

**Figure 4:**
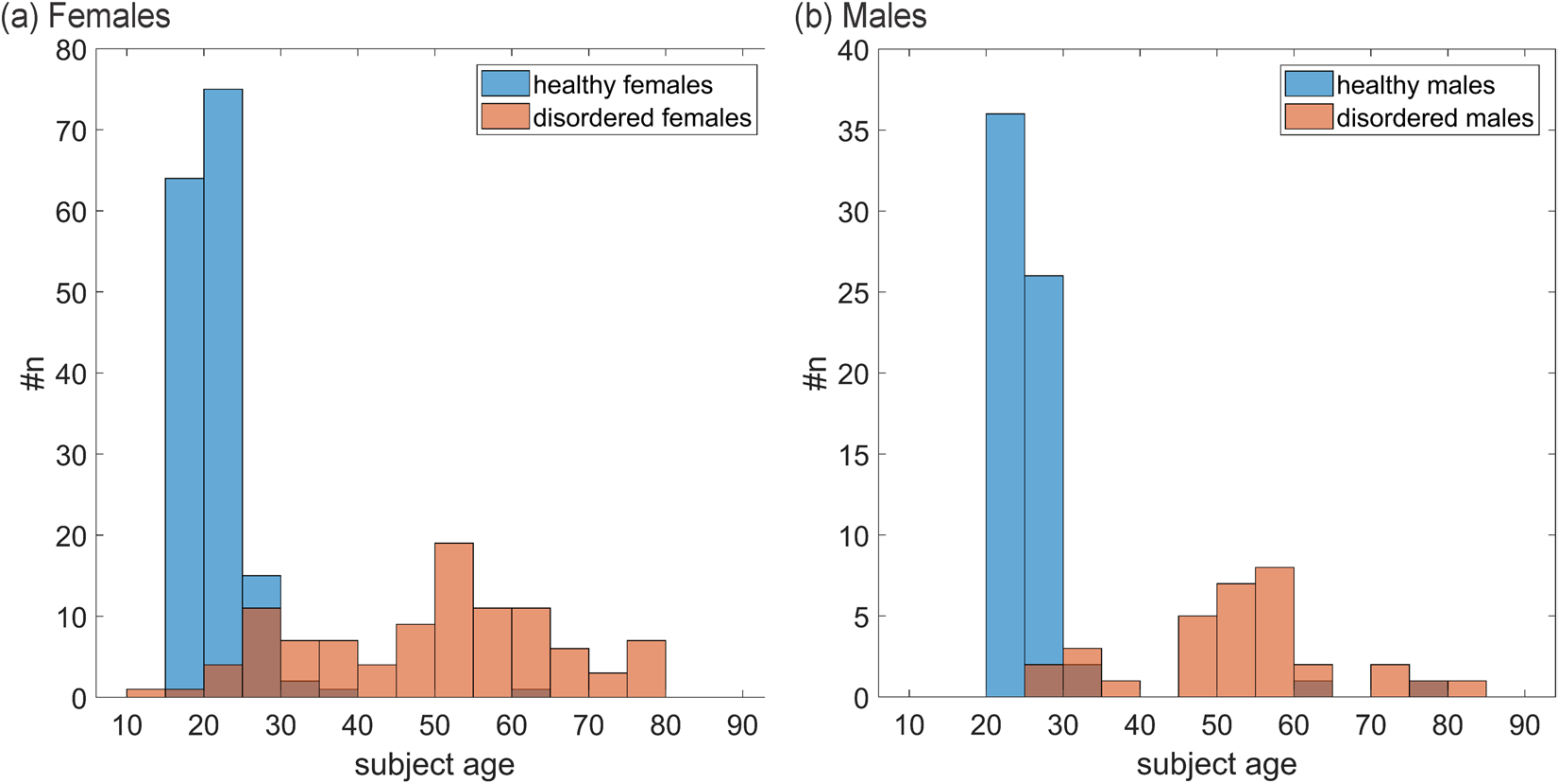
Distribution of subject age; for (**a**) females and (**b**) males for healthy and disordered groups with #n being the number of subjects.

A large difference in age between healthy and disordered groups exists. This is a common problem in clinical studies^37,38^. For this reason, it was investigated if subject age had a substantial influence on parameters for females and males. In Fig. 4, the age distribution of the healthy and disordered subjects is shown for females and males.

PCCs between each parameter and the age of the subjects for the groups FD_F_ and FD_M_ were calculated. The influence of subject age was investigated only for disordered subjects since all healthy subjects had a similar age (see Fig. 4). Furthermore, the p-value and a confidence interval of each PCC were calculated using the Matlab function “corrcoef”^39^. The p-value states if the correlation is statistically significantly different from zero (alpha = 0.05). The confidence interval calculated with Matlab is an estimator of the 95% confidence interval of the calculated PCC (see^39^). In this way a statement regarding the degree of linear dependencies between parameters and age can be made.

Following the suggestions of Mukaka a correlation was seen as negligible if it was 0.3 or lower^36^. Only a little number of “low” correlations (between 0.3 and 0.5^36^) were detected and no PCC was higher than 0.5. For this reason, the influence of subject age on this data was seen as negligible. Also, non-linear dependencies were investigated by reviewing scatter-plots of the parameter values against subject age but no obvious relations were found.

### Model selection and optimization

Exclusion of redundant parameters yielded parameter set HSV_1_. Now, two group comparisons were used for classification:

1. N_F_ vs. FD_F_
2. N_M_ vs. FD_M_

For each comparison, models applying the supervised learning classification approach of single level boosted trees (also: boosted stumps) were generated. This approach uses trees consisting of one node and two leaves each for data separation. After each added tree stump, data weights are recalculated allowing the separation of otherwise hardly separable subjects (this process is called “boosting”)^40,41^. We decided to use boosted stumps, since they performed comparatively well for the separation of a range of different data sets using various classification performance measures in comparison to other classification algorithms^42^. However, to avoid overfitting we decided to use boosted stumps instead of boosted trees, which achieved best overall performance in class separation^41,42^. For all models the “name value pair arguments” of the MATLAB function “fitcensemble”^43^ that was used for model generation were set as follows:

1. ‘prior’ was set to ‘uniform’ because of imbalanced class sizes,
2. ‘surrogate’ was set to ‘off’ since no data was missing,
3. ‘MaxNumSplits’ was set to 1 to avoid overfitting (i.e. trees consisted of only one node),
4. ‘LearningRate’ was set to 0.1 for training with shrinkage to find a better optimum.

For performance measure calculation, ten-fold cross validation was used. To prevent influences by random partitioning, each model was recalculated ten times. All performance measures were averaged over testing partitions and recalculated models. In the following **five steps**, it will be shown how the models were generated:

**Step 1 - Determine the boosting algorithm best suited for this problem:** Three boosted decision stumps algorithms “AdaBoost”, “LogitBoost” and “RUSBoost” were investigated. “AdaBoost” was included since it is one of the most widely applied boosting algorithms and hence a common choice^41^. “LogitBoost” is an algorithm designed for hardly separable classes and “RUSBoost” is designed for unbalanced class sizes^43^. Both are the case for our data.

Algorithm performance was rated using the performance measures, area under curve (AUC) and accuracy (ACC) (the higher the better). These measures complement each other to some degree. ACC can be misleadingly high for unbalanced class sizes but AUC is not influenced by class sizes. On the other hand, AUC can be misleadingly low for extremely sharply separated classes. However, for the final models, also sensitivity and specificity are given to show that no class is overly preferred^44^.

Further, it was investigated how much these algorithms weighted two added random parameters (a normal and an equally distributed variable) by measuring feature importance (FI). FI is a measure that states how important each feature (i.e. parameter) is for group separation (for more details see^45^). Therefore it is expected that the two added random parameters only achieve low importance. If random parameters would achieve high FI the algorithm would be unsuitable for this investigation.

**Step 2 - Determine the number of decision stumps to include in the model:** Applying the best algorithm determined in **Step 1**, models consisting of one up to 500 consecutive tree stumps were generated (without random parameters). AUC and ACC were plotted over the number of included stumps, i.e. model complexity. Based on these plots, an optimal number of stumps was chosen for the following models.

**Step 3 - Find the parameters that achieve the best result in separating N**_**F**_ **vs. FD**_**F**_**:** The FI for HSV_1_ parameters was determined for the group comparison N_F_ vs. FD_F_. Afterwards, models (as many as remaining parameters) were generated: The first of these models included only the parameter that was rated most important by FI, the second model included the parameter that was rated most important and the parameter rated second most important by FI and the last model included all parameters. From these models, one model was selected that achieved high AUC and ACC with only a small number of parameters. The parameters included in this model were rated as best set of parameters for this model comparison.

**Step 4 - Find the parameters that achieve the best result in separating N**_**M**_ **vs. FD**_**M**_: Analogous to Step 3 but for the group comparison N_M_ vs. FD_M_

**Step 5 - Find the combined parameter set that best separates female and male group comparisons:** Models including different combinations of the parameters found in **Step 3** and **Step 4** were generated. Investigation of all possible combinations was not feasible. Therefore, only certain combinations (e.g. only PVG or GAW based parameters) were investigated. A final parameter set (**HSV**_**2**_) that achieved the best compromise between high performance measures and a low number of parameters for both comparisons N_F_ vs. FD_F_ and N_M_ vs. FD_M_ was determined.

## Results and Discussion

Parameters were reduced in two main steps yielding parameter sets **HSV**_**1**_ and **HSV**_**2**_. In the following the steps leading to these parameter sets and their possible applicability are discussed.

### Linear dependencies

Table 2 shows the parameters that were correlated “very high” (corr ≥ 0.9^36^). It is stated which of the parameters were kept and why. After discarding 25 of 91 parameters, the parameter set HSV_1_ consisting of 66 parameters remains. The 25 discarded parameters are marked in Table S1.

**Table 2:** Groups of redundant parameters (corr: ≥ 0:9) It is stated which of multiple parameters are kept and why. 25 out of 36 parameters were discarded. The *-symbol indicates that some of the correlations of this parameter in this group are marginally below 0.9 for some cases.

Correlated parameter values	Kept value	Reasoning
corr ≥ 0.9		
AP [Mean], AP [Std]*, APQ3, APQ5, APQ11, MShim, APF	MShim	Widely applied, straightforward
TP [Mean], Jit(%), PPQ3, PPQ5, PPQ11, PPF, RAP_K_	Jit(%)	Widely applied, straightforward
EPQ3, EPQ5 EPQ11, EPF	EPF	Unexpected behavior found for EPQ-based parameters in^5^
PhAI[Mean], WaSI[Mean]	PhAI[Mean]	Faster to calculate
AmSI[Std], AmS[Std]*, DyRSI[Std], DyRS[Std]*	AmSI[Std]	Consistent with^5^
PhA[Std], PhAI[Std]	PhAI[Std]	No risk of cancellation of inverse phase shifts
SpA[Std], SpAI[Std]	SpAI[Std]	Consistent with PhAI[Std]
CAS^CA^[Std], CASI^CA^[Std]	CASI^CA^[Std]	Otherwise possible under- estimation of asymmetry because of cancellation effects
0.9 > corr ≥ 0.7		
TP[Std], F0[Std]	F0[Std]	TP [Mean] already removed
DyRS[Mean], AmS[Mean]	AmS[Mean]	Consistent with^5^
DyRSI[Mean], AmSI[Mean]	AmSI[Mean]	Consistent with^5^

It is stated which of multiple parameters are kept and why. 25 out of 36 parameters were discarded. The *-symbol indicates that some of the correlations of this parameter in this group are marginally below 0.9 for some cases.

By only excluding parameters that were correlated very high across all subjects, a conservative approach on parameter reduction was taken. Since the correlation was consistently high, it is reasonable to assume that it is due to the mathematical similarity of the underlying parameters. Parameters contained in HSV_1_ may not be completely independent but all obviously superfluous parameters were removed.

### Influence of subject age

**Figure 5:**
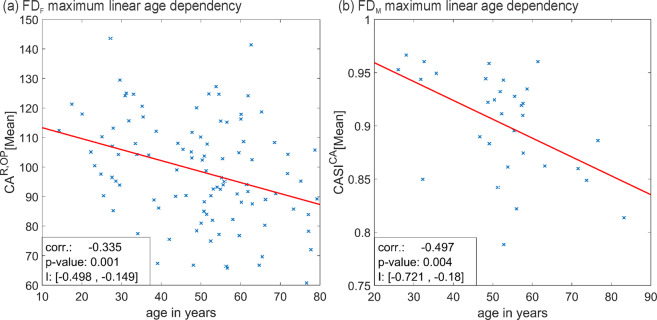
Parameters correlated highest with age; for (**a**) FD_F_ (**b**) FD_M_.

Influence of age was investigated for all HSV_1_ parameters. The calculated PCCs and estimated confidence intervals for groups FD_F_ and FD_M_ are listed in the supplementary information in Tables S2 and S3. Table S2 contains GAW-based parameters i.e. based on GAW_T_ (or GAW_L_ and GAW_R_ in case of symmetry measures). Table S3 contains PVG-based parameters. The highest absolute correlation values considering all parameters were −0.335 for CA^R,OP^ [Mean] in FD_F_ and −0.497 for CASI^CA^[Mean] in FD_M_. The scatter plots of these parameters for the respective groups against subject age are shown in Fig. 5. In addition, each plot contains a fitted line. Investigating the scatter plots in Fig. 5, no clear linear or nonlinear coherence between age and the depicted parameters is evident. Scatterplots of the remaining parameters were similar. Therefore, it was concluded that correlations of parameters with age are negligible for this study.

### Model selection and optimization

**Figure 6:**
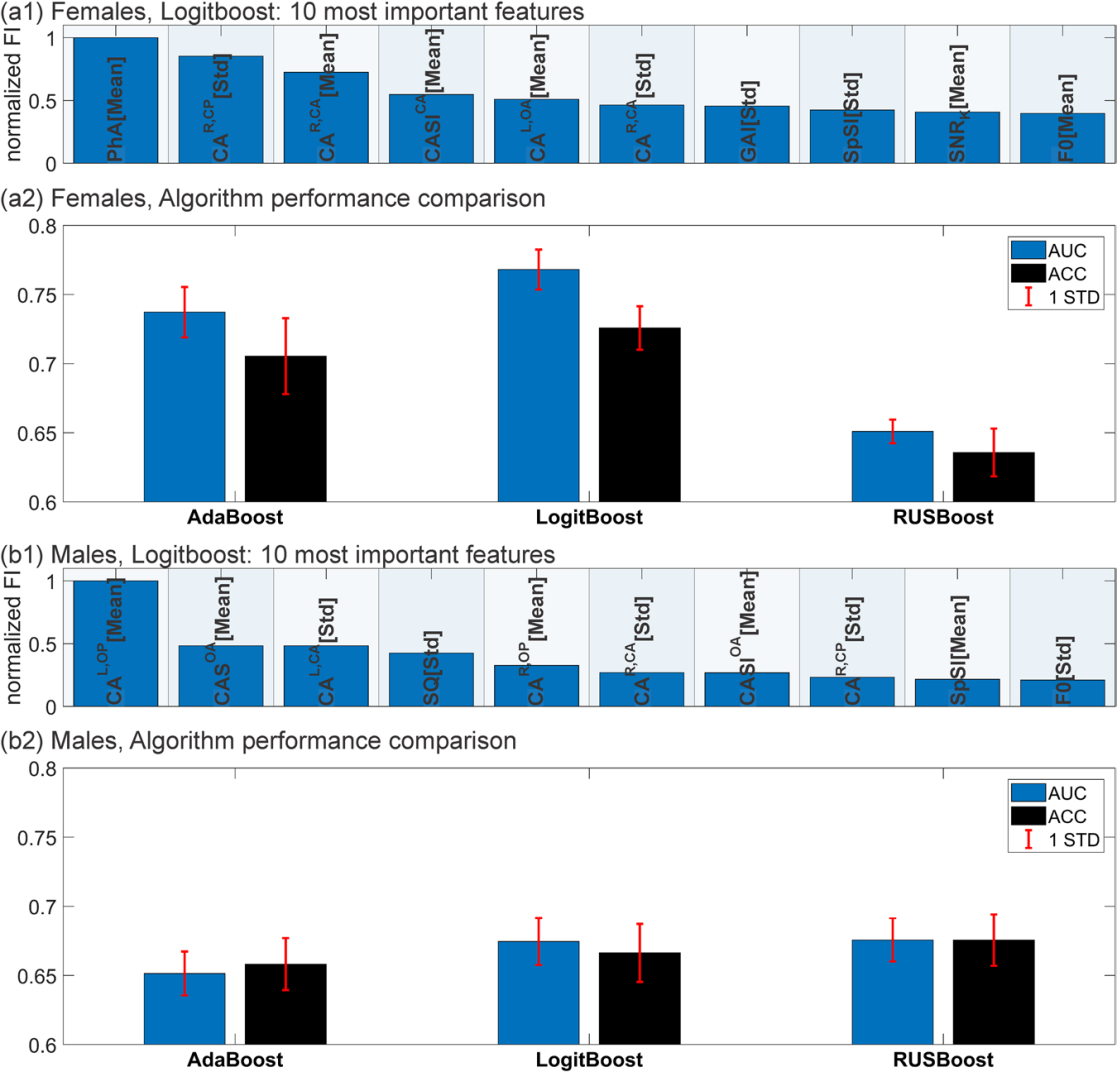
Comparison of boosting algorithms for (**a**) females and (**b**) males. (a1/b1) normalized feature importance of the 10 highest ranked parameters for Logitboost using a 300 stumps model. (a2/b2) comparison of AUC and ACC of all tested algorithms.

**Step 1:** The Algorithm judged as best was LogitBoost since it provided the highest AUC and ACC on average for both group comparisons (for models with and without added random parameters) and still did not rate the random parameters as important. This is also illustrated in Fig. 6 depicting (a1/b1) the normalized FI of the ten parameters rated most important and (a2/b2) average AUC and ACC for all three tested algorithms.

**Figure 7:**
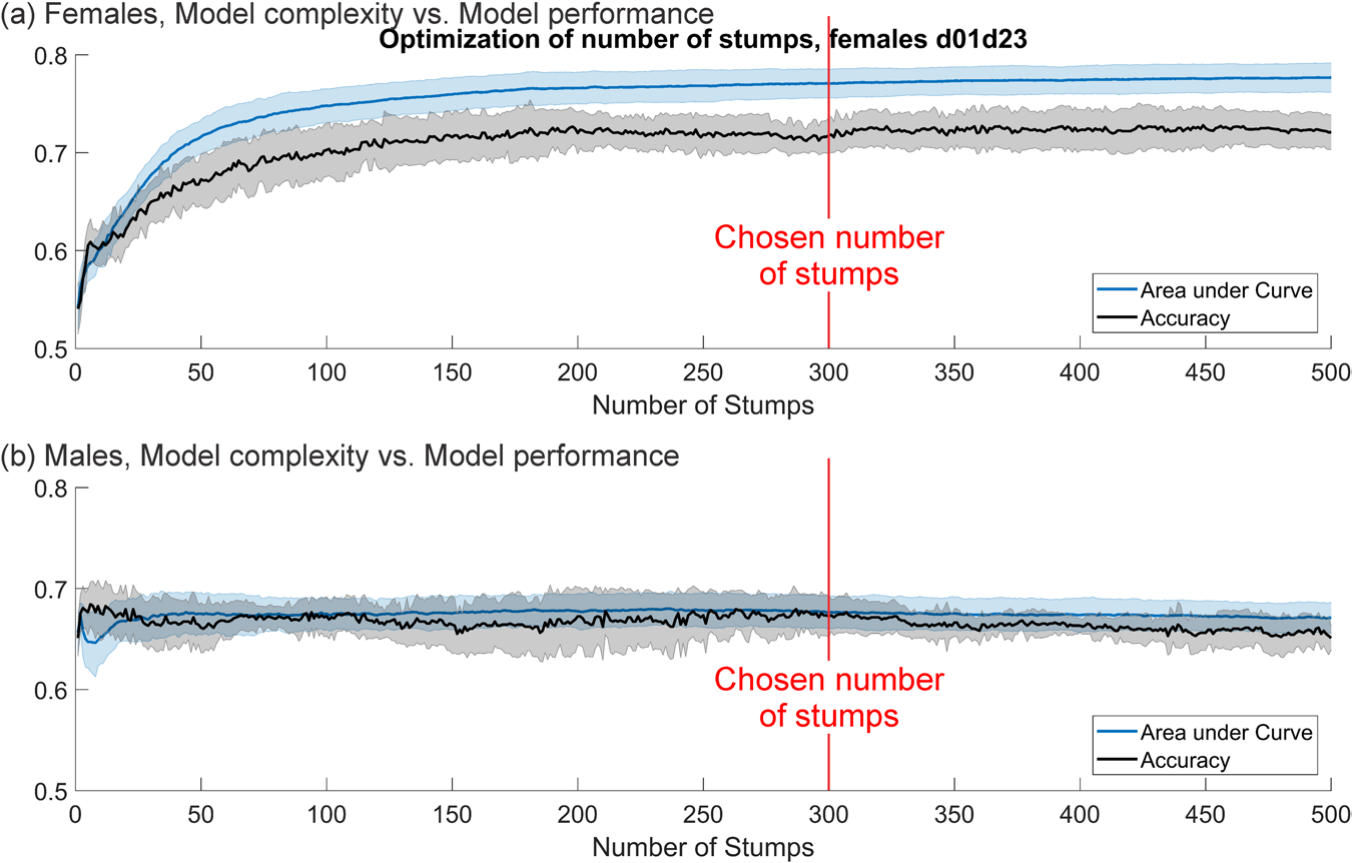
Choosing the optimal number of stumps for (**a**) females and (**b**) males. Number of stumps (model complexity) included in the model versus performance in measured in AUC and ACC.

**Step 2:** A number of 300 stumps was chosen for the following models, since neither for females nor for males AUC and ACC increase after approximately 300 stumps are reached (See Fig. 7).

**Figure 8:**
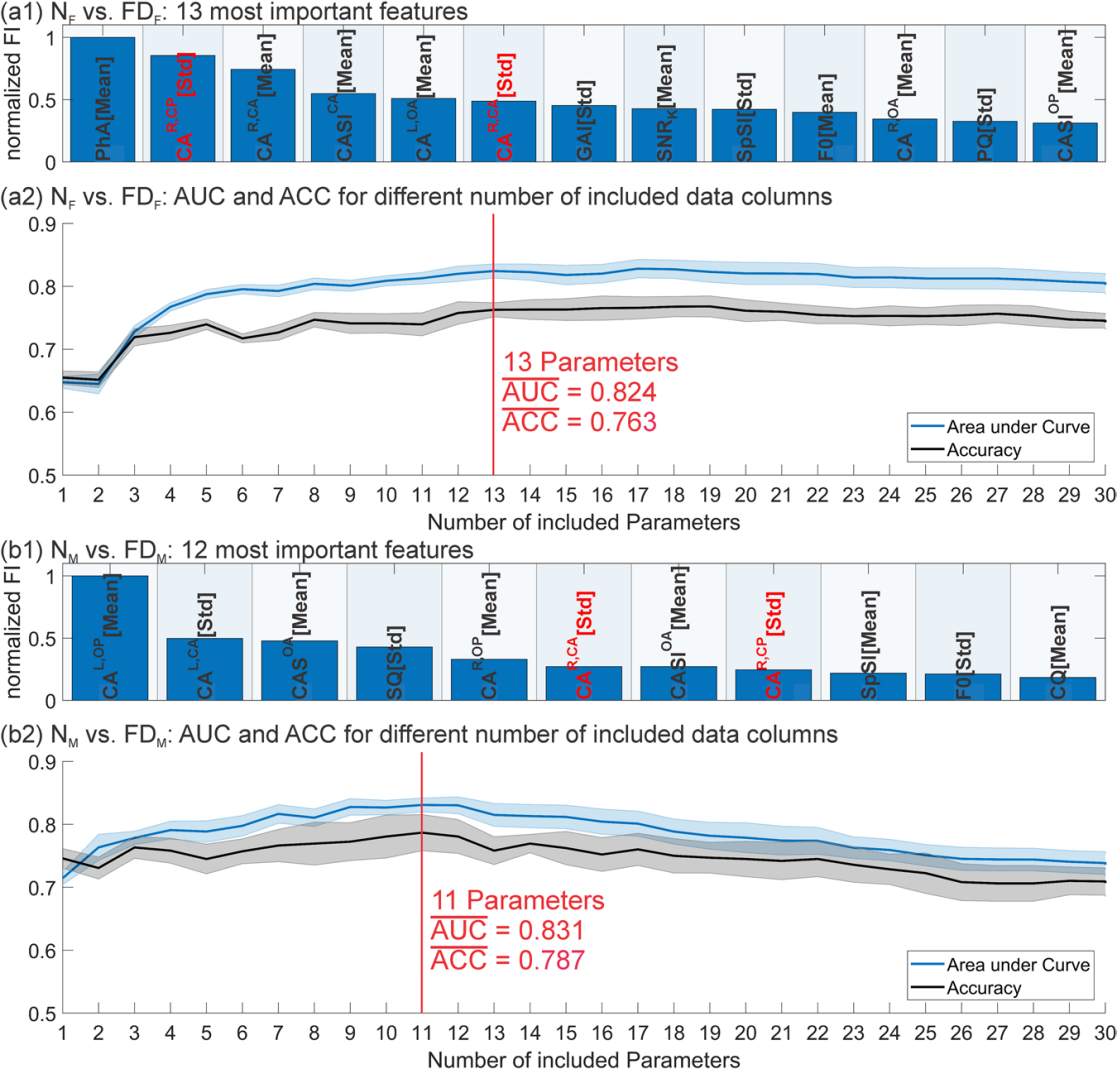
Determination of best parameter subset for group comparisons (**a**) N_F_ vs. FD_F_ and (**b**) N_M_ vs. FD_M_. (a1/b1) normalized FI of the 13/11 parameters ranked as most important. (a2/b2) AUC and ACC of models including only the best rated parameter, the best and the second best rated parameter,…. Parameters that were included in the best set for both group comparisons are marked in red.

**Step 3 and 4:** In Fig. 8, the results for group comparison N_F_ vs. FD_F_ and N_M_ vs. FD_M_ are depicted. Normalized FI of the parameters rated as most important for comparisons (a1) N_F_ vs. FD_F_ and (b1) N_M_ vs. FD_M_ are shown. For comparison (a2) N_F_ vs. FD_F,_ 13 parameters need to be included until AUC and ACC do not improve substantially anymore. Analogously, (b2) 11 parameters are included for N_M_ vs. FD_M_. Afterwards the model performance decreases. These parameters are respectively the 13 parameters in Fig. 8 (a1) and 11 parameters in Fig. 8 (b1). Since two parameters are included in both comparisons (marked in red), altogether 22 parameters were found to be relevant.

**Step 5:** In Table 3, AUC and ACC values of models for relevant parameter combinations are given for both group comparisons. The table also shows the number of included parameters reasoning which types of parameters were contained. The final parameter set HSV_2_, given in Table 4, was determined as best compromise between a still comparatively high AUC and ACC and a small number of included parameters. Average specificity (healthy subjects correctly identified as healthy) and sensitivity (disordered subjects correctly identified as disordered) for this set were 0.766 and 0.712 for group comparison N_F_ vs. FD_F_ and 0.767 and 0.772 for N_M_ vs. FD_M_. Average difference between specificity and sensitivity was 0.061 (N_F_ vs. FD_F_) and 0.088 (N_M_ vs. FD_M_). Therefore, no group was overly preferred.

**Table 3:** Relevant combinations of parameters and resulting AUC and ACC.

Number	Type of parameters	AUC females/males	ACC females/males
23	both parameter sets	0.812/0.771	0.752/0.722
13	only parameters found relevant in N_F_ vs. FD_F_	0.824/0.558	0.763/0.547
12	only parameters found relevant in N_M_ vs. FD_M_	0.694/0.831	0.647/0.787
13	only PVG-based parameters	0.770/0.763	0.713/0.756
10	only GAW-based parameters	0.716/0.599	0.686/0.597
12	Best parameter subset (HSV_2_)	0.788/0.804	0.745/0.768

**Table 4:** Final parameter set HSV_2_.

	GAW-based	PVG-based
parameter [Mean]	SNR_K_, PhA	CA^L, OP^, CA^R, CA^, CAS^OA^, CASI^OA^, CASI^CA^
parameter [Std]	SQ, PQ	CA^L, CA^, CA^R, CA^, CA^R, CP^

Table 5 contains mean and standard deviation of AUC and ACC for models using HSV_2_ and HSV_1_. The AUC of the model using HSV_2_ was on average 0.072 better than the model using the larger parameter set HSV_1_. The ACC was 0.061 better on average. In Table 6, mean values and standard deviations of all parameters assembled in HSV_2_ are given separated by groups (N_F_, FD_F_, N_M_ and FD_M_).

**Table 5:** Comparison of mean/standard deviation for AUC and ACC between parameter sets HSV_1_ and HSV_2_.

Group comparison	AUC	ACC
Females: HSV_2_ (12 parameters) vs. HSV_1_: (66 parameters)		
N_F_ vs. FD_F_	0.788/0.012 vs. 0.771/0.015	0.745/0.012 vs. 0.718/0.019
Males: HSV_2_ (12 parameters) vs. HSV_1_: (66 parameters)		
N_M_ vs. FD_M_	0.804/0.014 vs. 0.676/0.017	0.768/0.029 vs. 0.673/0.020
Average over all group comparisons		
Averaged	0.796/0.013 vs. 0.724/0.016	0.757/0.021 vs. 0.696/0.020

**Table 6:** Mean and standard deviation of groups N_F_, FD_F_, N_M_ and FD_M_.

		N_F_		FD_F_		N_M_	FD_M_
Mean/standard deviation							
PVG-based							
CA^L, OP^ [Mean] (°)	100.8/12.9		99.9/15.8		96.9/17.2		80.9/16.3
CA^R, CA^ [Mean] (°)	87.6/5.9		87.7/9.4		83.1/9.1		78.5/8.6
CAS^OA^ [Mean] (a.u.)	0.976/0.141		1.001/0.154		0.995/0.084		1.020/0.103
CASI^OA^ [Mean] (a.u.)	0.883/0.070		0.880/0.073		0.933/0.045		0.923/0.049
CASI^CA^ [Mean] (a.u.)	0.934/0.035		0.904/0.061		0.921/0.044		0.902/0.048
CA^L, CA^ [Std] (°)	3.4/1.7		4.3/3.0		2.9/1.6		4.0/1.7
CA^R, CA^ [Std] (°)	3.4/1.8		4.4/2.1		3.1/1.5		3.3/1.0
CA^R, CP^ [Std] (°)	6.6/4.3		6.5/5.1		5.3/6.5		2.9/2.1
GAW-based							
SNR_K_ [Mean] (dB)	11.2/1.4		10.5/1.6		11.1/1.3		11.0/1.4
PhA [Mean] (a.u.)	−0.031/0.080		0.001/0.113		−0.001/0.078		−0.011/0.092
SQ [Std] (a.u.)	0.151/0.065		0.174/0.085		0.155/0.057		0.165/0.100
PQ [Std] (a.u.)	0.047/0.011		0.052/0.014		0.043/0.013		0.051/0.018

HSV_2_ was able to clearly outperform the larger parameter set HSV_1_ even though all parameters included in HSV_2_ are also assembled in HSV_1_. This means that most parameters in HSV_1_ do not provide valuable information for group separation and only complicate the distinction. However, even the best achieved accuracies never exceeded 0.8. This implies that not all information that is needed for a definite distinction between healthy and disordered subjects is represented by the investigated parameters.

In the final parameter set HSV_2_, the GAW based parameters are underrepresented. This is especially noticeable since in HSV_1_, GAW based parameters were in the majority (GAW: 36 to PVG: 30). The indication that GAW based parameters may be less important than PVG based ones can also be concluded from Table 3. Including only GAW based parameters from the combined set yielded distinctly less AUC and ACC than including only PVG based parameters, especially for males. Since disordered voices are generally associated with aperiodic oscillations^13–15^, the GAW, as a measure exclusively of the glottal area, may not be sufficient to describe all features of such irregular vocal fold oscillations. Furthermore, by compressing the entire actual 3D-information of the vocal fold motion^46–48^ into a 1D-GAW-signal, much information is lost. In the PVG, the information is only compressed in 2D-space meaning less information loss in comparison to the GAW.

The initial parameter set HSV_1_ found for the group comparison N_F_ vs. FD_F_ did not perform well for the group comparison N_M_ vs. FD_M_ and vice versa (see Table 3). This illustrates the considerable difference in vocal fold dynamical characteristics between females and males.

**Figure 9:**
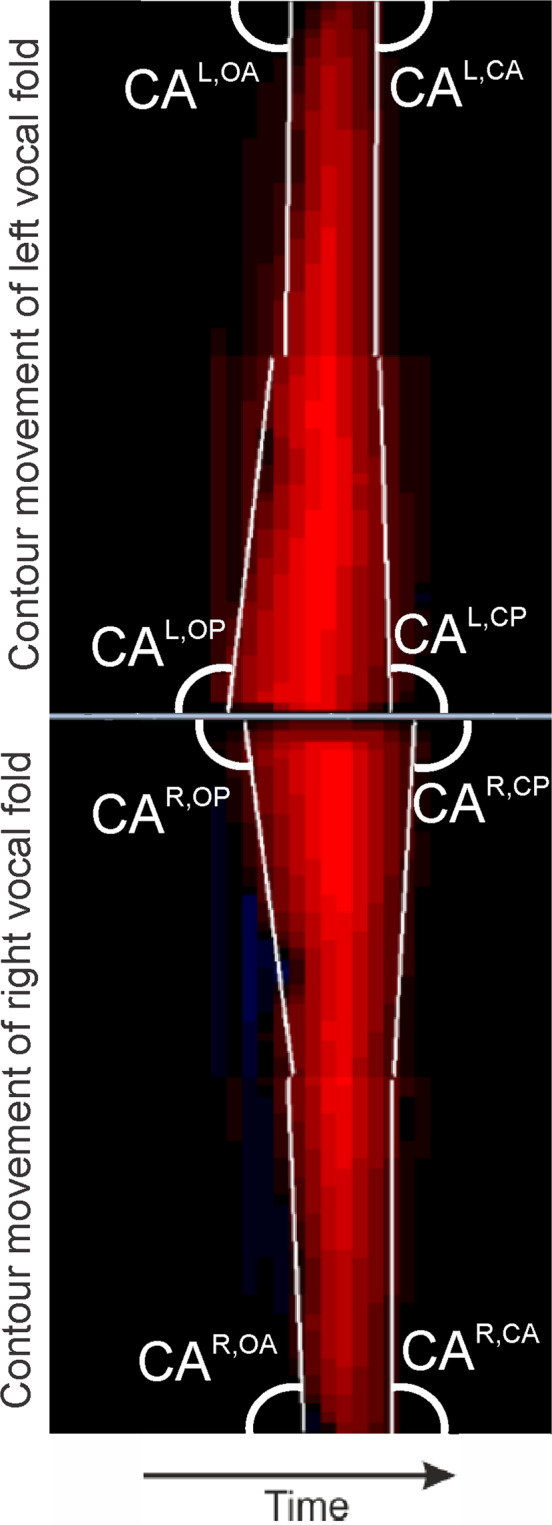
PVG oscillation cycle of healthy vocal folds with indicated contour angles.

The final subset HSV_2_ performed as well as the gender combined subset of 22 parameters and in some cases even better (see Table 3). The parameter set HSV_2_ consists of four types of parameters:

**Type 1:** Phonovibrogram (**PVG)** contour angle measures and contour angle symmetry measures. Different contour angles describe if the glottis opens or closes from anterior to posterior direction or vice versa and how fast this process is (see Table S1). For instance, a contour angle CA^L,OA^ [Mean] of 90° means that the left vocal fold (L) on its anterior half during opening phase (OA) opens simultaneously from the anterior part until its middle part. All CAS and CASI measures describe the symmetry of left and right pairs of contour angles. The different contour angles are illustrated in Fig. 9. Contour angle measures and contour angle symmetry measures describe roughly the oscillation pattern of the vocal folds. Therefore, it seems natural that they play the most important role in differentiating between normal and FD groups.

**Type 2:** SNR_K_ [Mean] is the only **noise measure** included in HSV_2_. It describes the relative energy of the harmonics in relation to the total energy of the signal in the Fourier spectrum^49^. A higher value implies a greater proportion of harmonics in the total spectrum and, as can be seen in Table 6, the GAWs of healthy subjects seem to be slightly more “harmonic” on average.

**Type 3:** The **symmetry measure** PhA [Mean] describes if the oscillations of the left and right vocal folds are in phase or time shifted. In the healthy case, this measure is expected to be close to zero. PhA [Mean] is a mean value. This means that positive and negative phase shifts in different cycles will cancel each other out. However, there is also a parameter that measures the absolute phase shift (PhAI [Mean]) which would not cancel out during averaging. This parameter was in no case selected as relevant by the boosting algorithm. This could be a hint that time-shifted vocal fold oscillations are only associated with FD if the time-shift is consistent.

**Type 4:** Standard deviations of two glottal dynamic characteristic parameters (SQ [Std] and PQ [Std]) were selected. These parameters describe the ratio between closing and opening phase and the “peakiness” of the GAW_T_^50,51^. The fact, that the standard deviations and not the average values of these parameters were selected, indicates that the actual shape of a GAW_T_ seems to be not as important as that this shape is consistent over time (i.e. cycles). Also in Table 6, the mean values of these parameters are slightly higher for the disordered cases. This means that SQ and PQ change more strongly on average between cycles for disordered subjects.

### Summary

From 91 investigated HSV-parameters (HSV_0_) only 12 parameters (HSV_2_, 13%) were required to separate healthy and FD subjects with fair accuracy of 74.5% respectively 76.8%. This final parameter set HSV_2_ also outperformed parameter set HSV_1_ (consisting out of 66 parameters). This indicates a large number of unneeded parameters for this separation task. However, no accuracies exceeding 0.8 could be achieved, hinting that not the entire information needed is contained in these parameters. Accuracies found in this work are mostly on a par with literature values of 0.76^28^ and 0.75^29^ for similar tasks. One study achieved a slightly better performance of 0.81 accuracy using only PVG-based measures^30^. Since in this study not the same PVG-based features were investigated as in our study, this may explain the difference. However, performance measures also varied considerable between recalculated models with different partitioning, so the observed difference may also be explainable purely by chance. The main gains from this investigation are the following:

1. 25 of the investigated 91 parameters are highly redundant (see section Linear dependencies in Results and Discussion and Table 2).
2. GAW-based parameters are less suited in differentiation healthy and FD subjects than PVG-based parameters. However, they provide valuable additional information.
3. Average values and standard deviations of parameters are both relevant. Regularity of GAW phases (SQ) and peak shape (PQ), harmonic structure (SNR_K_) and regularity and average values of different contour angles are mainly important.

### Shortcomings

Only parameters based on HSV-recordings were investigated. Other recording techniques, like stroboscopy or videokymography, were not applied. It is possible that better performance in separating healthy and FD subjects could have been achieved if more parameters from more signal sources, e.g. simultaneously recorded audio, would have been investigated in this work.

Due to the different age ranges of the healthy and the disordered group, results could have been influenced by subject age. An influence of subject age for different signal types and voice parameters is well documented in the literature^52–54^. However, in this study this influence should be low or even negligible as the variations in the data caused by FD seemed to outclass the influence of subject age by far.

Finally, more parameters, alternating parameter definitions and signal types exist that were not investigated in this study. However, with the investigation of 91 different parameters, we covered a large partition of the HSV parameters in use in voice research^24^.

## Conclusion

In this study we derived the subset HSV_2_ of 12 relevant HSV-parameters (mean of SNR_K_, PhA, CA^L, OP^, CA^R, CA^, CAS^OA^, CASI^OA^, CASI^CA^ and standard deviation of SQ, PQ, CA^L, CA^, CA^R, CA^, CA^R, CP^) from a set of 91 parameters (HSV_0_). Parameters in HSV_2_ reflected FD induced impairments and were sufficient to separate healthy and FD subjects with fair accuracy. The high degree of redundancy within parameters is shown by (1) exclusion of 25 parameters from HSV_0_ due to very high correlations yielding HSV_1_ and (2) 12 parameters in HSV_2_ even outperforming 66 parameters in HSV_1_ during group separation. Sources for investigated parameters can be found here:^55–70^.

Furthermore, this work shows that PVG-based parameters may be more relevant for differentiation between healthy and FD subjects than GAW-based parameters. However, best results were achieved by a combination of both. Also, the combination of boosted stumps and the FI measure were confirmed as a reliable approach to find relevant parameters and it was shown that the influence of subject age on our results is negligible.

This study affirms the need of multidimensional approaches for assessment of clinical data. Single parameters based on single signal sources are not sufficient to identify disorders. However, a too large amount of parameters also negatively affects results. By finding the best set of parameters, clinically applicable tools could be created assisting in assessment and therapy judgement of voice disorders. This could significantly objectify and improve current clinical routine.

## Supplementary information

Supplementary Information.
