# Supplementary material for: Machine learning based identification of relevant parameters for functional voice disorders derived from endoscopic high-speed recordings
Source: Sci Rep. 2020 Jun 29;10:10517. doi: 10.1038/s41598-020-66405-y (PMC7324600; doi:10.1038/s41598-020-66405-y)
Supplement: Supplementary file 1 — Supplementary Information. [file 41598_2020_66405_MOESM1_ESM.pdf]

Supplementary Information

Machine learning based identification of relevant parameters for functional voice disorders derived from endoscopic high-speed recordings

Patrick Schlegel[1*], Stefan Kniesburges[1], Stephan Dürr[1], Anne Schützenberger[1], Michael Döllinger[1]

[1] Dep. of Otorhinolaryngology, Div. of Phoniatrics and Pediatric Audiology, University Hospital Erlangen, Friedrich-Alexander-University Erlangen-Nürnberg, Erlangen, Germany

*Corresponding author:

Email: patrickschlegel93@yahoo.de

Table S1: Parameter information with parameters found to be highly redundant highlighted in blue. The table was split in sub tables for better readability.

Parameter (unit) and reference	Abbreviation	Parameter description
**GAW-based measures**		
**1. Fundamental period measures (FPM)**		
Mean of Fundamental Frequency (Hz)	*F0[Mean]*	reciprocal of cycle duration (Averaged for all cycles)
Standard deviation (Std) of Fundamental Frequency (Hz)	*F0[Std]*	reciprocal of cycle duration (Std for all cycles)

**2. Period perturbation measures (PPM)**		
Mean of Time Periodicity (a.u.) [55]	*TP[Mean]*	Deviation in duration between cycle pairs (Averaged for all cycle pairs)
Std of Time Periodicity (a.u.) [55]	*TP[Std]*	Deviation in duration between cycle pairs (Std for all cycle pairs)
Mean Jitter (ms) [56]	*MJit*	Mean deviation in duration between cycle pairs
Jitter (%)(a.u.) [56]	*Jit(%)*	Normalized mean deviation in duration between cycle pairs
Period Perturbation Quotient-3% (a.u) [57][1]	*PPQ3*	Difference in cycle lengths based on the mean difference between all inner cycles and two neighboring cycles
Period Perturbation Quotient-5% (a.u.) [57][1]	*PPQ5*	Difference in cycle lengths based on the mean difference between all inner cycles and four neighboring cycles
Period Perturbation Quotient-11% (a.u.) [57][1]	*PPQ11*	Difference in cycle lengths based on the mean difference between all inner cycles and ten neighboring cycles
Period Perturbation Factor (a.u.) [57][1]	*PPF*	Mean normalized deviation in duration between cycle pairs
Relative Average Perturbation $_{Koike}$ (a.u.) [58]	RAP_K	Normalized difference in cycle lengths based on the difference between all inner cycles and two neighboring cycles
Period Variability Index (a.u.) [59]	*PVI*	Normalized mean quadratic deviation in duration between each cycle and an average cycle

3. Amplitude perturbation measures (APM)		
Mean of Amplitude Periodicity (a.u.) [55]	*AP[Mean]*	Deviation in dynamic range between cycle pairs (Averaged for all cycle pairs)
Std of Amplitude Periodicity (a.u.) [55]	*AP[Std]*	Deviation in dynamic range between cycle pairs (Std for all cycle pairs)
Mean Shimmer (dB) [Horii] [56]	*MShim*	Mean logarithmic deviation in dynamic range between cycle pairs
Amplitude Perturbation Quotient-3% (a.u.) [57][1]	*APQ3*	Difference in dynamic range based on the mean difference between all inner cycles and two neighboring cycles
Amplitude Perturbation Quotient-5% (a.u.) [57][1]	*APQ5*	Difference in dynamic range based on the mean difference between all inner cycles and four neighboring cycles
Amplitude Perturbation Quotient-11% (a.u.) [57][1]	*APQ11*	Difference in dynamic range based on the mean difference between all inner cycles and ten neighboring cycles
Amplitude Perturbation Factor (a.u.) [57][1]	*APF*	Mean normalized deviation in dynamic range between cycle pairs

4. Energy perturbation measures (EPM)		
Energy Perturbation Quotient-3% (a.u.) [57] [1]	*EPQ3*	Difference in energy based on the mean difference between all inner cycles and two neighboring cycles
Energy Perturbation Quotient-5% (a.u.) [57][1]	*EPQ5*	Difference in energy based on the mean difference between all inner cycles and four neighboring cycles
Energy Perturbation Quotient-11% (a.u.) [57][1]	*EPQ11*	Difference in energy based on the mean difference between all inner cycles and ten neighboring cycles
Energy Perturbation Factor (a.u.) [57][1]	*EPF*	Mean normalized deviation in energy between cycle pairs

**5. Symmetry measures (SM)**		
Mean of Phase Asymmetry Index (a.u.) [60]	*PhAI[Mean]*	Mean of cyclewise difference in phase of GAW_L and GAW_R (side independent) $\lvert$position of minimum of GAW_L - position of minimum of $GAW_R$$\rvert$ / cycle length (side independent) [2]
Mean of Phase Asymmetry (a.u.) [60]	*PhA[Mean]*	Mean of cyclewise difference in phase of GAW_L and GAW_R (position of minimum of GAW_L - position of minimum of GAW_R) / cycle length
Mean of Spatial Symmetry Index (a.u.) [60]	*SpSI[Mean]*	Mean of cyclewise difference in area of GAW_L and GAW_R (side independent)[2]
Mean of Spatial Symmetry (a.u.) [60]	*SpS[Mean]*	Mean of cyclewise difference in area of GAW_L and GAW_R
Mean of Amplitude Symmetry Index (a.u.)[3]	*AmSI[Mean]*	Mean of cyclewise difference in maximal glottal area of GAW_L and GAW_R (side independent)[2]
Mean of Amplitude Symmetry (a.u.) [60]	*AmS[Mean]*	Mean of cyclewise difference in maximal glottal area of GAW_L and GAW_R
Mean of Dynamic Range Symmetry Index (a.u.) [60]	*DyRSI[Mean]*	Mean of cyclewise difference in dynamic range of GAW_L and GAW_R (side independent) [2]
Mean of Dynamic Range Symmetry (a.u.) [60]	*DyRS[Mean]*	Mean of cyclewise difference in dynamic range of GAW_L and GAW_R
Mean of Waveform Symmetry Index (a.u.) [60]	*WaSI[Mean]*	Mean of cyclewise difference in overall shape of GAW_L and GAW_R (side independent)[2]
Std of Phase Asymmetry Index (a.u.) [60]	*PhAI[Std]*	Std of cyclewise difference in phase of GAW_L and GAW_R (side independent) $\lvert$position of minimum of GAW_L - position of minimum of $GAW_R$$\rvert$ / cycle length (side independent) [2]
Std of Phase Asymmetry (a.u.) [60]	*PhA[Std]*	Std of cyclewise difference in phase of GAW_L and GAW_R (position of minimum of GAW_L - position of minimum of GAW_R) / cycle length
Std of Spatial Symmetry Index (a.u.) [60]	*SpSI[Std]*	Std of cyclewise difference in area of GAW_L and GAW_R (side independent)[2]
Std of Spatial Symmetry (a.u.) [60]	*SpS[Std]*	Std of cyclewise difference in area of GAW_L and GAW_R
Std of Amplitude Symmetry Index (a.u.)[3]	*AmSI[Std]*	Std of cyclewise difference in maximal glottal area of GAW_L and GAW_R (side independent)[2]
Std of Amplitude Symmetry (a.u.) [60]	*AmS[Std]*	Std of cyclewise difference in maximal glottal area of GAW_L and GAW_R
Std of Dynamic Range Symmetry Index (a.u.) [60]	*DyRSI[Std]*	Std of cyclewise difference in dynamic range of GAW_L and GAW_R (side independent) [2]
Std of Dynamic Range Symmetry (a.u.) [60]	*DyRS[Std]*	Std of cyclewise difference in dynamic range of GAW_L and GAW_R
Std of Waveform Symmetry Index (a.u.) [60]	*WaSI[Std]*	Std of cyclewise difference in overall shape of GAW_L and GAW_R (side independent)[2]

6. Glottal dynamic characteristics (GDC)		
Mean of Closing Quotient (a.u.) [61]	*CQ [Mean]*	Glottis closing time/ cycle duration (Averaged for all cycles)
Mean of Speed Quotient (a.u.) [50]	*SQ [Mean]*	Glottis opening time/ glottis closing time (Averaged for all cycles)
Mean of Glottis Gap Index (a.u.) [62][4]	*GGI [Mean]*	Minimum glottal area/maximum glottal area (Averaged for all cycles)
Mean of Plateau Quotient (a.u.) [51]	*PQ [Mean]*	Duration during which the glottal area has more than 95% of its maximum/cycle duration (Averaged for all cycles)
Mean of Glottal Area Index (a.u.) [63]	*GAI [Mean]*	Dynamic range/(maximum of glottal area times Open Quotient) (Averaged for all cycles)
Std of Closing Quotient (a.u.) [61]	*CQ [Std]*	Glottis closing time/ cycle duration (Std for all cycles)
Std of Speed Quotient (a.u.) [50]	*SQ [Std]*	Glottis opening time/ glottis closing time (Std for all cycles)
Std of Glottis Gap Index (a.u.) [62][4]	*GGI [Std]*	Minimum glottal area/maximum glottal area (Std for all cycles)
Std of Plateau Quotient (a.u.) [51]	*PQ [Std]*	Duration during which the glottal area has more than 95% of its maximum/cycle duration (Std for all cycles)
Std of Glottal Area Index (a.u.) [63]	*GAI [Std]*	Dynamic range/(maximum of glottal area times Open Quotient) (Std for all cycles)

7. Noise measures (NM)		
Cepstral Peak Prominence (dB) [64]	*CPP*	Describes the difference between the height of the fundamental quefrency peak and the value of a regression line fitted to the cepstrum at the same position.
Harmonics-to-Noise Ratio (dB) [65]	*HNR*	Describes how similar all cycles of a signal are to an averaged cycle.
Max. Waveform Matching Coefficient (a.u.) [66]	WMC_{Max}	Calculates similarity between neighboring sections of the signal of equal length. Gives back maximum similarity
Mean Waveform Matching Coefficient (a.u.) [66]	WMC_{Mean}	Calculates similarity between neighboring sections of the signal of equal length. Gives back average similarity
Normalized Noise Energy (dB) [67]	*NNE*	Estimates a pure noise signal using sections of the Fourier transformed input signal between the harmonics. Afterwards the Fourier transformed input signal is set in relation to the Fourier transformed pure noise signal.
Mean of Signal-to-Noise $\text{Ratio}_{\text{Klingholz}}$ (dB) [49]	SNR_K *[Mean]*	Creates an artificial pure harmonic signal in the Fourier domain based on the harmonics in the input signal. Afterwards the spectral energy of the pure harmonic signal and the spectral energy of the input signal are set in relation. (Average for all windows)
Signal-to-Noise Ratio_{Qi} (dB) [68]	SNR_Q	Estimates a noise signal based on residual signals derived from the input signal via linear prediction filtering. Afterwards the input signal and the noise signal are set in relation.
Std of Signal-to-Noise $\text{Ratio}_{\text{Klingholz}}$ (dB) [49]	SNR_K *[Std]*	Analogous to SNR_K *[Mean]* (Std for all windows)

PVG-based measures	
**8. Contour Angles**	
Mean of Left anterior opening contour angle (°)[5] [69]	$CA^{L,OA}[Mean]$
Mean of Left posterior opening contour angle (°) [69]	$CA^{L,OP}[Mean]$
Mean of Left anterior closing contour angle (°)[69]	$CA^{L,CA}[Mean]$
Mean of Left posterior closing contour angle (°)[69]	$CA^{L,CP}[Mean]$
Mean of Right anterior opening contour angle (°)[69]	$CA^{R,OA}[Mean]$
Mean of Right posterior opening contour angle (°)[69]	$CA^{R,OP}[Mean]$
Mean of Right anterior closing contour angle (°) [69]	$CA^{R,CA}[Mean]$
Mean of Right posterior closing contour angle (°) [69]	$CA^{R,CP}[Mean]$
Mean of Contour angle sym. between OA-angles (a.u.) [70][6]	$CAS^{OA}[Mean]$
Mean of Contour angle sym. between OP-angles (a.u.) [70]	$CAS^{OP}[Mean]$
Mean of Contour angle sym. between CA-angles (a.u.) [70]	$CAS^{CA}[Mean]$
Mean of Contour angle sym. between CP-angles (a.u.) [70]	$CAS^{CP}[Mean]$
Mean of Cont. ang. sym. Index between OA-angles (a.u.) [70]	$CASI^{OA}[Mean]$
Mean of Cont. ang. sym. Index between OP-angles (a.u.) [70]	$CASI^{OP}[Mean]$
Mean of Cont. ang. sym. Index between CA-angles (a.u.) [70]	$CASI^{CA}[Mean]$
Mean of Cont. ang. sym. Index between CP-angles (a.u.) [70]	$CASI^{CP}[Mean]$
Std of Left anterior opening contour angle (°)[69]	$CA^{L,OA}[Std]$
Std of Left posterior opening contour angle (°)[69]	$CA^{L,OP}[Std]$
Std of Left anterior closing contour angle (°)[69]	$CA^{L,CA}[Std]$
Std of Left posterior closing contour angle (°)[69]	$CA^{L,CP}[Std]$
Std of Right anterior opening contour angle (°)[69]	$CA^{R,OA}[Std]$
Std of Right posterior opening contour angle (°)[69]	$CA^{R,OP}[Std]$
Std of Right anterior closing contour angle (°)[69]	$CA^{R,CA}[Std]$
Std of Right posterior closing contour angle (°) [69]	$CA^{R,CP}[Std]$
Std of Contour angle sym. between OA-angles (a.u.) [70]	$CAS^{OA}[Std]$
Std of Contour angle sym. between OP-angles (a.u.) [70]	$CAS^{OP}[Std]$
Std of Contour angle sym. between CA-angles (a.u.) [70]	$CAS^{CA}[Std]$
Std of Contour angle sym. between CP-angles (a.u.) [70]	$CAS^{CP}[Std]$
Std of Cont. ang. sym. Index between OA-angles (a.u.) [70]	$CASI^{OA}[Std]$
Std of Cont. ang. sym. Index between OP-angles (a.u.) [70]	$CASI^{OP}[Std]$
Std of Cont. ang. sym. Index between CA-angles (a.u.) [70]	$CASI^{CA}[Std]$
Std of Cont. ang. sym. Index between CP-angles (a.u.) [70]	$CASI^{CP}[Std]$

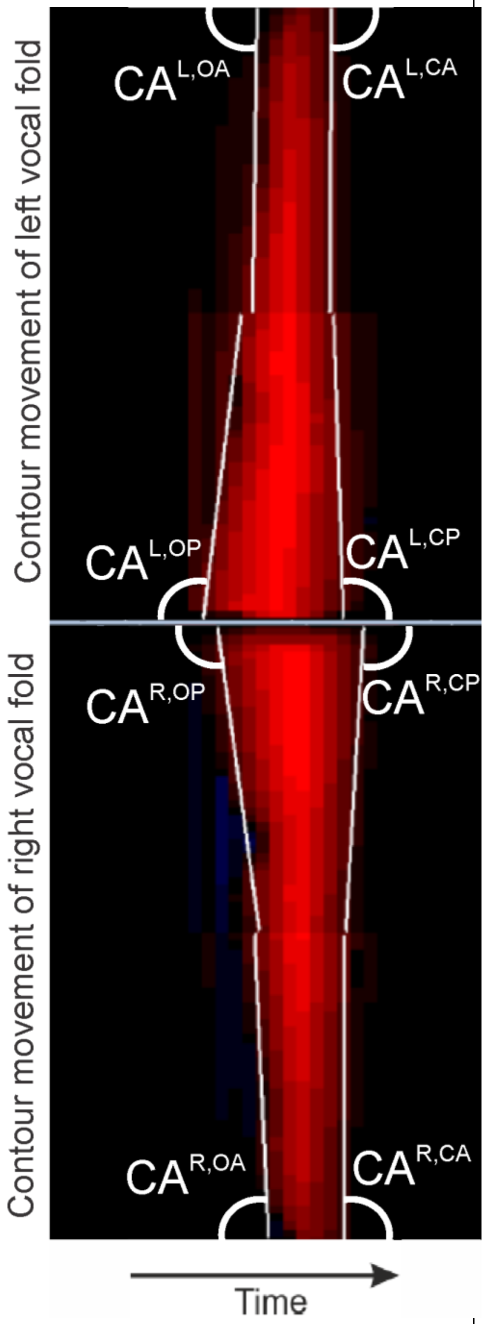

[1] in the source material one formula is given as “Perturbation Quotient” and one as “Perturbation Factor”. The different types of Perturbation Quotients and Factors in this work were calculated by inserting cycle lengths, dynamic ranges and cycle energies in these original formulas for, in case of the Perturbation Quotient, values of k of 3, 5 and 11. [2] Side dependent versions of symmetry measures give information about the direction of an asymmetric behavior. However, since they are calculated cycle-wise and then averaged over all cycles, left and right-sided asymmetries can cancel each other out. For this reason, side independent versions of the measures exist. They can give information about the actual size of an asymmetric effect, but not about the direction (See also [59]). [3]AmSI = (min(max[left glottal area], max[right glottal area]))/ (max(max[left glottal area], max[right glottal area]))) [4] Derived from the “Glottis Closure Index” in the source material. [5] All contour angles are calculated cycle-wise as shown on the right image. Average values and Stds of all angels of one type calculated over one video are investigated. [6] In the source material CAS^{OA} is named as "Ratio_OA", analogous for other contour angle symmetries.

Table S2: Pearson correlation coefficients (PCC)s including confidence intervals calculated between GAW-based parameters and subject age. A *-symbol indicates a statistically significant correlation.

	FD_F		FD_M	
Parameter name	PCC	confidence interval	PCC	confidence interval
Fundamental period measures (FPM)				
F0 [Mean]	-0.069	[-0.261, 0.128]	0.150	[-0.210, 0.474]
F0 [Std]	0.020	[-0.176, 0.215]	0.053	[-0.302, 0.394]
Period, amplitude and energy perturbation measures (PPM, APM and EPM)				
MJitt	-0.024	[-0.218, 0.173]	-0.193	[-0.507, 0.167]
Jit(%)	-0.041	[-0.235, 0.156]	-0.155	[-0.477, 0.205]
PVI	0.046	[-0.151, 0.239]	-0.034	[-0.379, 0.318]
MShim	0.001	[-0.195, 0.196]	0.044	[-0.310, 0.387]
EPF	-0.140	[-0.327, 0.057]	-0.311	[-0.595, 0.043]
Symmetry measures (SM)				
PhAI [Mean]	0.173	[-0.023, 0.356]	-0.103	[-0.436, 0.255]
SpSI [Mean]	-0.078	[-0.269, 0.120]	-0.033	[-0.377, 0.32]
AmSI [Mean]	0.051	[-0.146, 0.244]	0.389*	[0.047, 0.650]
PhA [Mean]	0.122	[-0.075, 0.310]	-0.067	[-0.406, 0.289]
SpS [Mean]	-0.083	[-0.274, 0.115]	-0.090	[-0.426, 0.267]
AmS [Mean]	0.005	[-0.191, 0.200]	0.171	[-0.189, 0.491]
PhAI [Std]	0.041	[-0.156, 0.235]	0.198	[-0.162, 0.511]
SpSI [Std]	0.117	[-0.08, 0.306]	0.007	[-0.342, 0.355]
AmSI [Std]	0.079	[-0.119, 0.270]	-0.166	[-0.487, 0.194]
WaSI [Std]	0.104	[-0.094, 0.293]	-0.236	[-0.541, 0.123]
Glottal dynamic characteristics (GDC)				
CQ [Mean]	0.007	[-0.188, 0.203]	0.253	[-0.105, 0.553]
SQ [Mean]	-0.113	[-0.302, 0.084]	-0.229	[-0.535, 0.130]
GGI [Mean]	0.042	[-0.155, 0.236]	0.187	[-0.173, 0.503]
PQ [Mean]	0.246*	[0.053, 0.421]	-0.061	[-0.401, 0.294]
GAI [Mean]	0.075	[-0.123, 0.266]	-0.152	[-0.476, 0.208]
CQ [Std]	0.169	[-0.027, 0.353]	-0.012	[-0.359, 0.338]
SQ [Std]	0.047	[-0.149, 0.241]	-0.193	[-0.508, 0.167]
GGI [Std]	0.029	[-0.167, 0.223]	0.214	[-0.145, 0.524]
PQ [Std]	0.076	[-0.122, 0.267]	-0.141	[-0.467, 0.218]
GAI [Std]	0.166	[-0.03, 0.350]	0.140	[-0.220, 0.466]
Noise measures (NM)				
HNR	-0.051	[-0.244, 0.146]	0.041	[-0.312, 0.384]
NNE	0.144	[-0.053, 0.330]	-0.079	[-0.416, 0.278]
SNR_K [Mean]	-0.091	[-0.282, 0.106]	-0.108	[-0.440, 0.250]
SNR_Q	0.012	[-0.184, 0.207]	0.148	[-0.212, 0.472]
CPP	0.049	[-0.148, 0.243]	0.007	[-0.342, 0.355]
WMC_{max}	0.171	[-0.026, 0.354]	0.102	[-0.256, 0.435]
WMC_{mean}	0.018	[-0.178, 0.213]	0.196	[-0.164, 0.510]
SNR_K [Std]	-0.061	[-0.253, 0.137]	-0.105	[-0.438, 0.253]

Table S3: Pearson correlation coefficients (PCC)s including confidence intervals calculated between PVG-based parameters and subject age. A *-symbol indicates a statistically significant correlation.

	FD_F		FD_M	
Parameter name	PCC	confidence interval	PCC	confidence interval
Phonovibrogram measures (PVGM)				
$CA^{L, OA}$ [Mean]	0.120	[-0.077, 0.308]	0.026	[-0.326, 0.371]
$CA^{L, OP}$ [Mean]	-0.243*	[-0.418, -0.05]	-0.311	[-0.595, 0.042]
$CA^{L, CA}$ [Mean]	0.047	[-0.15, 0.240]	-0.029	[-0.374, 0.323]
$CA^{L, CP}$ [Mean]	-0.240*	[-0.416, -0.047]	0.051	[-0.303, 0.393]
$CA^{R, OA}$ [Mean]	0.090	[-0.107, 0.281]	0.107	[-0.251, 0.439]
$CA^{R, OP}$ [Mean]	-0.335*	[-0.498, -0.149]	-0.385*	[-0.647, -0.042]
$CA^{R, CA}$ [Mean]	-0.030	[-0.224, 0.166]	-0.287	[-0.578, 0.068]
$CA^{R, CP}$ [Mean]	-0.175	[-0.358, 0.021]	-0.015	[-0.361, 0.336]
CAS^{OA} [Mean]	0.072	[-0.125, 0.264]	-0.134	[-0.461, 0.225]
CAS^{OP} [Mean]	0.140	[-0.057, 0.327]	0.253	[-0.105, 0.553]
CAS^{CA} [Mean]	0.118	[-0.08, 0.306]	0.285	[-0.07, 0.577]
CAS^{CP} [Mean]	-0.072	[-0.263, 0.126]	0.090	[-0.267, 0.425]
$CASI^{OA}$ [Mean]	-0.217*	[-0.396, -0.023]	-0.038	[-0.382, 0.315]
$CASI^{OP}$ [Mean]	-0.065	[-0.257, 0.132]	-0.116	[-0.447, 0.242]
$CASI^{CA}$ [Mean]	-0.301*	[-0.469, -0.112]	-0.497*	[-0.721, -0.180]
$CASI^{CP}$ [Mean]	0.103	[-0.095, 0.292]	0.014	[-0.337, 0.361]
$CA^{L, OA}$ [Std]	0.145	[-0.052, 0.331]	0.074	[-0.282, 0.412]
$CA^{L, OP}$ [Std]	-0.113	[-0.301, 0.085]	-0.283	[-0.575, 0.073]
$CA^{L, CA}$ [Std]	0.100	[-0.098, 0.290]	0.263	[-0.094, 0.561]
$CA^{L, CP}$ [Std]	-0.178	[-0.361, 0.019]	-0.328	[-0.607, 0.023]
$CA^{R, OA}$ [Std]	0.201*	[0.005, 0.381]	-0.014	[-0.361, 0.336]
$CA^{R, OP}$ [Std]	0.076	[-0.121, 0.268]	-0.186	[-0.502, 0.174]
$CA^{R, CA}$ [Std]	0.240*	[0.047, 0.416]	0.385*	[0.042, 0.647]
$CA^{R, CP}$ [Std]	-0.063	[-0.256, 0.134]	-0.274	[-0.568, 0.083]
CAS^{OA} [Std]	0.166	[-0.031, 0.350]	0.281	[-0.075, 0.573]
CAS^{OP} [Std]	0.015	[-0.181, 0.210]	-0.109	[-0.441, 0.249]
CAS^{CP} [Std]	-0.141	[-0.327, 0.056]	-0.312	[-0.596, 0.042]
$CASI^{OA}$ [Std]	0.132	[-0.066, 0.319]	0.287	[-0.069, 0.578]
$CASI^{OP}$ [Std]	0.008	[-0.188, 0.203]	-0.038	[-0.381, 0.315]
$CASI^{CA}$ [Std]	0.193	[-0.002, 0.375]	0.330	[-0.021, 0.609]
$CASI^{CP}$ [Std]	-0.212*	[-0.391, -0.017]	-0.265	[-0.561, 0.093]
